# Phase 1 Study Evaluating the Association of the Cyclin-Dependent Kinase 4/6 Inhibitor Ribociclib and Cetuximab in Recurrent/Metastatic p16-Negative Squamous Cell Carcinoma of the Head and Neck

**DOI:** 10.3389/fonc.2019.00155

**Published:** 2019-03-19

**Authors:** Emmanuel Seront, Sandra Schmitz, Matthias Papier, Aline van Maanen, Stéphanie Henry, Christophe Lonchay, Sylvie Rottey, Gabrielle van Caloen, Jean-Pascal Machiels

**Affiliations:** ^1^Department of Medical Oncology, Centre Hospitalier de Jolimont, La Louvière, Belgium; ^2^Departments of Medical Oncology and Head and Neck Surgery, Institut Roi Albert II, Institut de Recherche Clinique et Expérimentale (Pole MIRO), Cliniques Universitaires Saint-Luc, Catholic University of Louvain, Woluwe-Saint-Lambert, Belgium; ^3^Statistical Support Unit, Institut Roi Albert II, Cliniques Universitaires Saint-Luc, Woluwe-Saint-Lambert, Belgium; ^4^Department of Medical Oncology, Centre de Maternité Sainte Elisabeth, Namur, Belgium; ^5^Department of Medical Oncology, Grand Hôpital de Charleroi, Charleroi, Belgium; ^6^Department of Medical Oncology, University Hospital Ghent, Ghent, Belgium; ^7^Laboratory of Medical Oncology, Institut Roi Albert II, Institut de Recherche Clinique et Expérimentale (Pole MIRO), Catholic University of Louvain, Namur, Belgium

**Keywords:** ribociclib, cetuximab, HPV, recurrent, squamous cell carcinoma, head and neck

## Abstract

**Background:** The majority of human papillomavirus (HPV)-negative squamous cell carcinoma of the head and neck (SCCHN) present upregulation of the epidermal growth factor receptor (EGFR) and frequent alterations in the cyclin D1-cyclin dependent kinase (CDK) 4/6 (CDK 4/6)-retinoblastoma protein (pRb) pathway, resulting in cell cycle progression and tumor proliferation. This study investigated the combination of ribociclib, an orally highly selective inhibitor of CDK 4/6, and cetuximab in recurrent and/or metastatic (R/M) SCCHN.

**Methods:** A phase I trial using a 3 + 3 design was performed to determine the dose limiting toxicity (DLT) and maximum tolerated dose (MTD) of ribociclib with standard dose of weekly cetuximab in HPV-negative patients with R/M SCCHN. Ribociclib was administered orally (3 weeks on/1 week off) at dose level 1 of 400 mg daily and dose level 2 of 600 mg daily. The MTD of ribocilib was then further evaluated in an expansion cohort.

**Results:** 10 patients were enrolled in the escalation trial. No DLTs were observed at dose level 1 (*n* = 3); at dose level 2, one patient was replaced due to rapid disease progression, and one patient out of six evaluable patients experienced a DLT (grade 4 thrombocytopenia >7 days). Ribociclib 600 mg daily was thus determined to be the MTD. Eleven additional patients were enrolled in the expansion cohort. Diarrhea (52%), rash (52%), fatigue (43%), nausea (33%), and mucositis (28%) were the most frequent grade 1–2 adverse events (AE). Neutropenia was the most frequent grade 3–4 AE (20%). Median progression-free survival (PFS) was 3.5 months (range 0.4–17.3 months) and median overall survival (OS) was 8.3 months (range 0.4–24.1 months). Among the 19 radiologically evaluable patients, two (10.5%) achieved a partial response and 11 (58%) had stable disease.

**Conclusions:** The MTD of ribociclib is 600 mg daily when administered in combination with standard dose cetuximab for 3 weeks on and 1 week off. This combination was safe and showed efficacy. Further clinical trials should be conducted to evaluate the antitumor effects of this combination.

**Trial Information:** ClinicalTrials.gov: NCT02429089; Eudract number 2014-005371-83.

## Introduction

Squamous cell carcinoma of the head and neck (SCCHN) is a major cause of cancer-associated illness and death worldwide, with approximately 750,000 new cases diagnosed annually (1). Most patients present with loco-regionally advanced disease and are treated with multimodal treatment including surgery, chemotherapy, and/or radiotherapy. However, 50% of patients experience disease relapse with a median survival not exceeding 12–14 months (2).

The epidermal growth factor receptor (EGFR) is overexpressed in 90% percent of SCCHN and is associated with worse prognosis and resistance to radiotherapy (3, 4). Cetuximab, a monoclonal antibody targeting the EGFR, has been shown to improve outcome when combined with radiation and chemotherapy (5, 6). In recurrent or metastatic (R/M) SCCHN, the addition of cetuximab to first-line platinum-based chemotherapy increases progression-free survival (PFS) from 3.3 to 5.6 months and overall survival (OS) from 7.4 to 10.1 months (6); however, resistance inexorably occurs. The efficacy of cetuximab as monotherapy after failure of platinum-based chemotherapy is modest at best as the overall response rate (ORR) is only 13% and survival does not exceed 6 months (7). This low response rate, together with treatment resistance and the absence of correlation between EGFR expression and cetuximab efficacy, suggest that additional factors, such as cell-cycle regulation proteins, should be considered when targeting the EGFR pathway (8).

Cyclin D1 is encoded by *CCND1* and is a cell-cycle protein that regulates the G1-to-S phase transition through the formation of complexes with cyclin dependent kinases (CDKs), such as CDK 4 and 6. Upon mitogenic signals, this complex cyclin D1-CDK4/6 inactivates by phosphorylation of the retinoblastoma protein (pRb), a negative regulator of cell cycle progression. Inactivation of pRb releases the transcription factor E2F, inducing expression of the S-phase gene and stimulating cell proliferation. The cyclin D1-CDK4/6 complex is inhibited by p16^INK4A^, which is encoded by the gene *CDKN2A* (9, 10). SCCHN, and particularly HPV-negative tumors, are characterized by frequent alterations in the cyclin D1-CDK4/6-pRb pathway. In the Tumor Cancer Genome Analysis (TCGA), *CCND1* amplification was observed in 28% of SCCHN, with its frequency reaching 32% in HPV-negative tumors compared to only 6% in those that were HPV-positive. Moreover, up to 57% of HPV-negative SCCHN have inactivation of *CDKN2A* compared to 0% in HPV-positive tumors (11).

Overexpression of cyclin D1 and amplification of *CCND1* in SCCHN are associated with poor prognosis and resistance to cisplatin and EGFR inhibition (12, 13). Previously, EGFR activity had been observed to regulate cell-cycle progression via ERK1/2-dependent induction of *CCND1* (14). Furthermore, a recent study in HPV-negative patients showed a strong inverse correlation between expression of EGFR and pRb inactivation as well as between EGFR mRNA upregulation and CDK6 upregulation and amplification (15). Afatinib, or lapatinib combined with a CDK4/6 inhibitor, is synergistic in terms of cell viability reduction in HPV-negative cell lines (15).

Ribociclib is a selective CDK4/6 inhibitor which has demonstrated antitumor activity in preclinical and clinical studies in a wide variety of tumor types, including breast cancer (16). We performed a phase I trial to determine the maximum tolerated dose (MTD) of ribociclib combined with standard weekly doses of cetuximab in patients with R/M HPV-negative SCCHN.

## Materials and Methods

### Inclusion and Exclusion Criteria

Eligible patients had to have R/M HPV-negative SCCHN not amenable to curative treatment with surgery and/or chemotherapy and/or radiation; progressive disease (PD) within 1 year of first-line platinum-based chemotherapy given either as part of multimodal curative treatment or in the palliative setting; Eastern Cooperative Oncology Group performance status (ECOG PS) 0–1; at least one measurable lesion according to the Response Evaluation Criteria in Solid Tumors 1.1 (RECIST); and the ability to swallow ribociclib tablets. Previous treatment with cetuximab was allowed either in the metastatic or curative setting (with radiation). Patients needed to have adequate organ function, absolute neutrophil count (ANC) >1,500/mm^3^, hemoglobin ≥9 g/dl, platelet count >100,000/mm^3^, serum creatinine ≤1.5 the upper limit of normal (ULN), total bilirubin ≤1 ULN, and alanine aminotransferase (ALAT) and aspartate aminotransferase (ASAT) <1.5 ULN. HPV-negative tumor was defined by the absence of p16^INK4A^ staining. The study was approved by an independent ethics committee, the Belgian health authorities, and conducted in accordance with the Declaration of Helsinki (October 2000). Written informed consent was obtained for each patient.

### Study Objectives

The primary objective was to determine the MTD of ribociclib in combination with the recommended dose of cetuximab. The secondary objective was to evaluate the toxicity profile of ribociclib in combination with cetuximab in patients with R/M SCCHN. With exploratory intent, we also assessed treatment efficacy by reporting ORR [according to RECISTv1.1 (17)], PFS and OS. Tissue samples were collected for further translational analysis.

### Study Design

Cetuximab 400 mg/m^2^ was given intravenously on cycle 1 day 1, followed by 250 mg/m^2^ weekly, as per standard recommendations (18). Ribociclib was administered orally with food on days 1–21 of each 28-day cycle.

This phase I study used a classical 3 + 3 dose escalation design. The first dose level of ribociclib was defined as 400 mg/day and the second dose level as 600 mg/day; three patients were enrolled per dose level and intra-patient dose escalation was not permitted. If zero out of three patients experienced a dose limiting toxicity (DLT), the dose was escalated to the next dose level. When one out of three patients experienced a DLT, three additional patients had to be included. In the event that two out of three or six patients experienced a DLT, dose escalation had to be stopped. The MTD was defined as the dose below the dose level at which two patients out of three or six experienced DLTs. Given that the MTD of ribociclib monotherapy is 600 mg, it was not planned to escalate ribociclib above this dose (19). In the event that the combination of ribociclib 600 mg and cetuximab (standard dosage) was found to be safe, it was pre-planned that the MTD of ribociclib in combination would be defined as 600 mg/day. DLTs and adverse events (AEs) were determined according to the NCI-CTC version 4.0. DLTs were evaluated during cycle 1 (4 weeks) and were defined as: (a) grade 4 neutropenia lasting more than seven consecutive days, (b) grade 4 thrombocytopenia, (c) grade 3–4 neutropenia with fever, (d) any ribociclib-related grade 3–4 toxicities with the exception of sub-optimally treated nausea/vomiting/diarrhea, or brief (<72 h) grade three fatigue and grade three electrolyte disturbance resolving to grade 1 with 7 days of drug interruption.

Criteria to initiate subsequent cycles included an ANC ≥1,000/mm^3^, platelets ≥50,000/mm^3^, and non-hematologic toxicities ≤ grade 1. If these were not met, ribociclib was delayed for 1 week while cetuximab was continued. After a 2-week delay, ribociclib was discontinued. The dose of ribociclib was adjusted for selected AEs. A dose decrease by 200 mg/day was recommended for grade 4 neutropenia/thrombocytopenia, grade 3 neutropenia with infection/fever, grade ≥3 non-hematologic toxicity, or treatment delay >1 week due to persisting AE if recovery occurred within 2 weeks. At 400 mg, one dose reduction was allowed, while at 600 mg, two dose reductions were allowed. Patients who required more than two dose reductions were treated with cetuximab alone.

After identification of the MTD, 14 patients were initially planned to be enrolled in an expansion cohort to further confirm the safety of this combination.

Treatment emergent adverse events (TEAE) were defined as events that occurred after study treatment initiation, or those that worsened relative to the pre-treatment state.

### Tumor Response Assessment

The efficacy of the ribociclib-cetuximab combination was evaluated with exploratory intent. Tumor response assessment was performed every two cycles (8 weeks), according to RECIST v1.1 criteria. ORR was defined as the proportion of patients with a complete response (CR) or a partial response (PR). PFS was defined as the time interval between the date of enrolment and the date of disease progression or the date of death due to any cause. OS was defined as the time interval between the date of enrolment until death due to any cause, or until the date of last follow-up. Patients with unknown or missing response data were treated as non-responders.

### Statistical Methods

The number of patients for dose escalation was determined by the MTD and DLTs to a maximum of 12 patients. Expecting an ORR >20% with the combination, 14 patients were planned to be included in the expansion phase with a 95.6% chance of achieving at least one success. PFS and OS were also estimated according to the Kaplan-Meier algorithm. Patients without any event (progression or death) were censored at the date of last-follow-up.

## Results

### Patient Characteristics

Twenty-one patients (14 men and 7 women) from five Belgian centers were included between April 2015 and May 2017 in this trial. Ten patients were included in the escalation cohort and, due to slow recruitment and premature closure of the study, only 11 were enrolled in the expansion cohort. The median age was 61.1 years (range 33–80 years). Fourteen patients (67%) were previously treated with cetuximab. Baseline characteristics are described in Table 1.

**Table 1:** Patient characteristics.

**Baseline characteristics**	**Ribociclib + Cetuximab (*N* = 21)**
**Age (Years)**	
Median (range)	61 (33–80)
**Gender**	
Male	14 (67%)
Female	7 (33%)
**ECOG PS**	
0	6 (29%)
1	15 (71%)
**Smoking Status**	
>10 pack-year	19 (91%)
<10 pack-year	2 (9%)
**Primary Site**	
Oropharynx	4 (19%)
Oral cavity	9 (43%)
Hypopharynx	3 (14%)
Larynx	2 (10%)
Unknown primary	3 (14%)
**Tumor Grade At Diagnosis**	
Well-differentiated	6 (29%)
Moderately differentiated	9 (43%)
Poorly differentiated	3 (14%)
Unknown/missing	3 (14%)
**Location Of Relapse At Inclusion**	
Local and/or regional only	14 (67%)
Metastatic alone	3 (14%)
Loco-regional and metastatic	4 (19%)
**Primary Curative Treatment**	
Surgery	16 (76%)
Radiation therapy	19 (90.5%)
Chemotherapy	
Induction	0 (0%)
Concomitant to radiation therapy	13 (62%)
**p16 Status**	
Positive	0 (0%)
Negative	21 (100%)
**Previous Platinum-Based Chemotherapy**	
Curative	16 (62%)
Palliative	15 (71%)
**Number Of Previous Lines In Palliative Setting**	
None	7 (33%)
1	7 (33%)
2 or more	7 (33%)
**Previous Cetuximab Administration**	
Curative intent	10 (48%)
Recurrent/metastatic setting	4 (19%)

### Dose Limiting Toxicity and Maximum Tolerated Dose

Three patients were enrolled in dose level 1 (400 mg/day) of ribociclib and no DLT was observed. Seven patients were enrolled in the dose level 2 escalation phase (600 mg/day): one patient presented with rapid disease progression (within 7 days) and was replaced as he could not be evaluated for DLT assessment; another experienced a DLT with grade 4 thrombocytopenia. Ribociclib 600 mg daily for 3 weeks on and 1 week off with standard dose cetuximab was therefore considered to be the MTD.

### Safety

Table 2 shows the most frequent TEAEs that occurred in the 21 patients regardless of dose levels. Diarrhea (52%), rash (52%), fatigue (43%), nausea (33%), and mucositis (28%) were the most frequent grade 1–2 AEs. Neutropenia was the most frequent grade 3–4 AE (20%). No febrile neutropenia was observed, and no grade 5 toxicity was recorded.

**Table 2:** Treatment emergent adverse events (NCI-CTC version 4.0).

	**Treatment emergent adverse events (TEAEs)**	**Ribociclib and Cetuximab (*n* = 21)**
	**Grade 1–2**	**Grade 3–4**
Number of patients with any TEAEs	20 (95%)	9 (43%)
**Constitutional**		
Fatigue	9 (43%)	1 (4%)
Anorexia	3 (14%)	2 (9%)
Rash	11 (52%)	0 (0%)
Pruritis	3 (14%)	0 (0%)
Conjunctivitis	2 (9%)	0 (0%)
Headache	2 (9%)	0 (0%)
QTc interval prolongation	1 (4%)	0 (0%)
**GASTROINTESTINAL**		
Nausea	7 (33%)	0 (0%)
Vomiting	5 (24%)	0 (0%)
Diarrhea	11 (52%)	0 (0%)
Oral mucositis	6 (28%)	1 (4%)
Dysphagia	2 (9%)	0 (0%)
Heartburn	1 (4%)	0 (0%)
**HEMATOLOGICAL**		
Anemia	5 (24%)	1 (4%)
Thrombocytopenia	6 (28%)	3 (14%)
Neutropenia	6 (28%)	4 (20%)
Lymphopenia	0 (0%)	3 (14%)
**BIOLOGICAL**		
Hypomagnesemia	7 (33%)	1 (4%)
Hypocalcemia	3 (14%)	1 (4%)
Hypokalemia	2 (9%)	1 (4%)
Hypophosphatemia	4 (20%)	0 (0%)

The median number of administered cycles was two (range 2–4) in patients receiving ribociclib 400 mg (*n* = 3), and three (range 0–19) in patients receiving ribociclib 600 mg (*n* = 18). One patient stopped ribociclib treatment 3 days after initiation due to rapid disease progression; two patients interrupted ribociclib temporarily and restarted with the same dose (one interrupted treatment for 14 days due to grade 3 anemia, while the other interrupted for seven days due to QT interval increase at 481 ms occurring without concomitant use of medication with a known risk to prolong the QT interval); five patients had a dose reduction (one for grade 3 fatigue and thrombocytopenia, one for grade 3 thrombocytopenia and neutropenia, and three for grade 3 neutropenia). Eighteen patients stopped due to disease progression, one died during treatment due to septic shock not related to ribociclib, one decided to stop despite stable disease (SD), and one stopped after a long lasting remission.

### Efficacy

Median PFS was 3.5 months (range 0.4–17.3 months) and median OS was 8.3 months (0.4–24.1 months). The PFS rates at six and 12-months were 19 and 4.7%, and the corresponding OS rates were 71 and 33%, respectively. Four patients remained alive after a median follow-up of 9.2 months (range 0.25–39 months). PFS was similar between patients previously treated and untreated with cetuximab (100 and 106 days, respectively).

**Figure 1:**
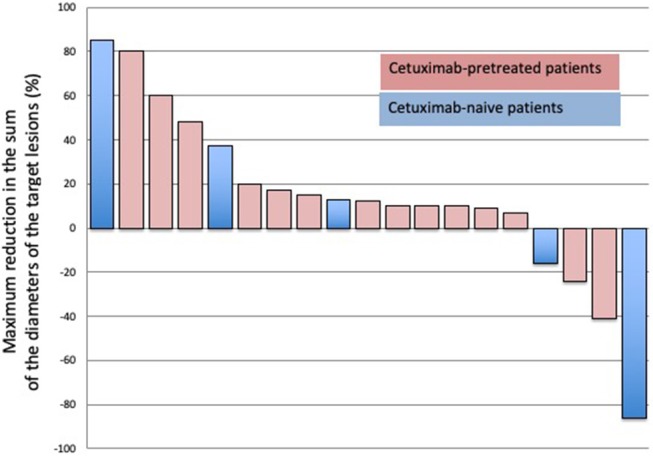
Waterfall plots showing the maximum percentage modification in the sum of the diameters of the target lesions in assessable patients.

Two patients were not evaluable for radiological evaluation; one patient progressed rapidly within 7 days of treatment initiation, and one died from septic shock not related to medication before the first radiological evaluation. Among the 19 evaluable patients, two (10.5%) achieved a PR, 11 (58%) had SD, and six (36%) had PD, resulting in a disease control rate (SD + PR) of 68.5%. Two of the 11 SD patients experienced a minor response with a maximum reduction in the sum of the diameters of the target lesions of −24 and −16% (Figure 1). From the 14 patients previously treated with cetuximab (10 with curative-intent and 4 in R/M setting), 10 presented with disease stabilization (including one with minimal reduction of the target lesion) and one experienced a partial response. This last patient was treated in a curative setting with cetuximab and the interval between last dose of cetuximab and study entry was 6 months. The four patients previously treated in R/M setting curative-intent presented a SD (without minor reduction of target lesions).

## Discussion

This trial is the first to establish the feasibility of combining the selective CDK4/6 inhibitor ribociclib with cetuximab in HPV-negative SCCHN. The escalation phase of this study identified 600 mg daily for 3 weeks on followed by 1 week off as the MTD of ribociclib in combination with cetuximab, which is also the recommended dose in metastatic breast cancer in association endocrine therapy (16).

The AEs observed in this trial were as expected for each drug, and the toxicity profile was consistent with a phase I trial that evaluated the association of palbociclib and cetuximab in SCCHN (20). Myelosupression was the main grade 3–4 AE related to ribociclib, manifesting principally as thrombocytopenia and neutropenia that resulted in dose reduction but not in definitive arrest. Gastrointestinal AEs were mostly mild and easily manageable. Fatigue and anorexia may have been related to ribociclib but are also frequently observed in heavily pretreated patients with advanced stage SCCHN.

We had to stop the trial early due to low accrual given that most of the investigators were opting to include their suitable patients in trials investigating anti-PD1 inhibitors. Although this trial was limited by small patient numbers, the combination of ribociclib and cetuximab resulted in an ORR of 10%, a disease control rate of 68%, a PFS of 3.5 months and an OS of 8.3 months. Interestingly, two patients pre-treated with cetuximab experienced some degree of tumor shrinkage. Recently, a phase II trial evaluated the efficacy of palbociclib combined with cetuximab in patients with platinum-resistant R/M SCCHN and reported a CR of 11% and PR of 29% for an ORR of 39%; median PFS reached 5.4 months and the median OS was 9.5 months (21). These results are promising and exceed, at least in terms of survival, those observed with nivolumab in the CheckMate 141 trial (22). However, this last study excluded patients previously treated with cetuximab for R/M setting and only 7% of the included patients had received cetuximab in a curative setting, which could explain the better results they observed compared to our trial (21). Indeed, our population included around 70% of patients who had previously been treated with cetuximab and this may have impaired treatment activity.

This phase I trial is the first to evaluate the combination of ribociclib and cetuximab in HPV-negative SCCHN. The recommended dose is 600 mg daily on a 3-weeks on and 1-week off schedule in combination with cetuximab at its standard dose. This combination proved safe. A larger phase II trial should be conducted in HPV-negative SCCHN patients to correctly evaluate the antitumor efficacy of this combination.

## Data Availability

All datasets generated for this study are included in the manuscript and/or the supplementary files.

## Ethics Statement

Ethic Committee from Cliniques universitaires Saint Luc (Comité dEthique Hospitalo-Facultaire de lUCL). Avenue Hippocrate 55.14- Tour Harvey; 1200 Brussels, Belgium. comission.ethique-saint-luc@uclouvain.be. Reference 2015/06JAN/005; Number 403 provided by Bioethic Consultatif Committee. Agreement of Institutional Review board: IRB 00001530 (Cliniques universitaires Saint-Luc) and IRB 00008535 (Université catholique de Louvain).

## Author Contributions

ES and J-PM wrote the body of the manuscript which was verified by all other authors. All authors contributed equally to the research.

### Conflict of Interest Statement

The authors declare that the research was conducted in the absence of any commercial or financial relationships that could be construed as a potential conflict of interest.
